# Sorting biotic and abiotic stresses on wild rocket by leaf-image hyperspectral data mining with an artificial intelligence model

**DOI:** 10.1186/s13007-022-00880-4

**Published:** 2022-04-02

**Authors:** Alejandra Navarro, Nicola Nicastro, Corrado Costa, Alfonso Pentangelo, Mariateresa Cardarelli, Luciano Ortenzi, Federico Pallottino, Teodoro Cardi, Catello Pane

**Affiliations:** 1 Council for Agricultural Research and Economics (CREA), Research Centre for Vegetable and Ornamental Crops, Via Cavalleggeri 25, 84098 Pontecagnano Faiano, Italy; 2 Consiglio per la Ricerca in Agricoltura e l’analisi dell’economia Agraria (CREA) - Centro di Ricerca Ingegneria e Trasformazioni Agroalimentari, Via della Pascolare 16, 00015 Monterotondo, Italy

**Keywords:** Hyperspectral imaging, Fusarium wilting, Machine learning, Rhizoctonia rotting, Water deficit, Salinity

## Abstract

**Background:**

Wild rocket (*Diplotaxis tenuifolia*) is prone to soil-borne stresses under intensive cultivation systems devoted to ready-to-eat salad chain, increasing needs for external inputs. Early detection of the abiotic and biotic stresses by using digital reflectance-based probes may allow optimization and enhance performances of the mitigation strategies.

**Methods:**

Hyperspectral image analysis was applied to *D. tenuifolia* potted plants subjected, in a greenhouse experiment, to five treatments for one week: a control treatment watered to 100% water holding capacity, two biotic stresses: Fusarium wilting and Rhizoctonia rotting, and two abiotic stresses: water deficit and salinity. Leaf hyperspectral fingerprints were submitted to an artificial intelligence pipeline for training and validating image-based classification models able to work in the stress range. Spectral investigation was corroborated by pertaining physiological parameters.

**Results:**

Water status was mainly affected by water deficit treatment, followed by fungal diseases, while salinity did not change water relations of wild rocket plants compared to control treatment. Biotic stresses triggered discoloration in plants just in a week after application of the treatments, as evidenced by the colour space coordinates and pigment contents values. Some vegetation indices, calculated on the bases of the reflectance data, targeted on plant vitality and chlorophyll content, healthiness, and carotenoid content, agreed with the patterns of variations observed for the physiological parameters. Artificial neural network helped selection of VIS (492–504, 540–568 and 712–720 nm) and NIR (855, 900–908 and 970 nm) bands, whose read reflectance contributed to discriminate stresses by imaging.

**Conclusions:**

This study provided significative spectral information linked to the assessed stresses, allowing the identification of narrowed spectral regions and single wavelengths due to changes in photosynthetically active pigments and in water status revealing the etiological cause.

**Supplementary Information:**

The online version contains supplementary material available at 10.1186/s13007-022-00880-4.

## Background

Plant diseases or abiotic stresses, such as water deficit or salinity, are key factors in the growth and yield of most vegetable crops [1–5]. The early detection and identification of both biotic and abiotic stresses would provide an opportunity for early intervention to control, prevent spread of infection or change irrigation management practices before the whole crop is damaged and vast yield losses occur [6]. Imaging sensors can identify the onset of adverse stresses before visible symptoms appear. Among imaging techniques, hyperspectral imaging is preferable for the identification and categorization of early stages of plant foliar diseases and abiotic stresses from laboratory to field scale, since uses high-fidelity plant reflectance information over a large range of the light spectrum, beyond that human vision, capturing more than the usual three bands of coloured light found in traditional digital imaging.

Hyperspectral imaging has already been used in vegetable crops for detecting biotic or abiotic stress, such as water salinity in lettuce [7], water stress in potato [8] and in tomato [9], Sclerotinia disease on oilseed rape [10], downy mildew in cucumber [11], most important diseases in tomato, bell pepper, potato and squash [12]. In order to determine the best combination of reflectance wavelengths sensitive for diagnosing water or saline stress as well as plant diseases, hyperspectral images can be studied and used on rocket leaves before visible damages create a high detriment value in the production of this vegetable primarily employed for the fresh consumption of a ready-to-eat product, due to its unique taste.

Wild or perennial rocket [*Diplotaxis tenuifolia* (L.) D.C.], is a leafy vegetable, commonly also known as arugula, roquette or rucola, belonging to the mustard family (Brassicaceae). It’s traditionally grown in the Mediterranean region, widely consumed in Italy, but with increasing popularity as green salad (mixed or alone) in other parts of the World thanks to its excellent nutritional properties and its antioxidant activity [13–17]. The major producer area of wild rocket in the European Union is the Southwest of Italy, with an annual cultivation area of about 4,800 hectares under protected cultivation with yields ranging between about 30–40 kg m^−2^ of fresh cut [18, 19]. Sustainability of the wild rocket productive processes must be increased in terms of reduction of synthetic fungicides applications for the effective soil-borne disease control, and to improve the water use efficiency. Moreover, water management, also regarding crop tolerance to salinity attributable to the concentration of salts in the highly fertigated soils or the use of low-quality irrigation water, is due. All these factors affect plant sap-flow efficiency, compromising plant vitality and then, yield.

*Fusarium oxysporum f. sp. raphani* Kendrick and Snyder is the causal agent of the wild rocket wilting [20], while *Rhizoctonia solani* Kühn [telomorph *Thanatephorus cucumeris* (Frank) Donk] is a parenchymatic pathogen causing rotting on roots, crown and collar [21]. Both telluric pathogens provoke dramatic epidemics under favourable environmental conditions (i.e., inoculum accumulation, high humidity, and continuous cropping of susceptible cultivars) like those that occur in sick and intensively exploited soils [22] and are the major soil-borne biotic adversities of the crop. Most Brassicas species have been categorized as moderately salt tolerant, with, however, a significant interspecific and intraspecific variation [23]. Contradictory findings exist regarding the reaction of these species to salt stress at different plant developmental stages, while most authors indicate that these species maintain their degree of salt tolerance consistently throughout the plant ontogeny. Regarding drought tolerance, although *Eruca vesicaria* has been reported to be one of the most drought-tolerant species in Cruciferae [24, 25] little is known about the ecology and physiology of *D. tenuifolia* under water deficit. *D. tenuifolia* could be considered as moderately drought tolerant, according to the results obtained by [26], where *D. tenuifolia* plants could cope with drought of 4 days without having any consequences on its growth, and the application of moderate-deficit irrigation (plants received 60% of the 100% crop evapotranspiration values) did not reduce plant growth and flower development.

So, to limit reduction of yields and avoid unexpected loss of earnings in the presence of the stress factors, the time-effective application of plant protection as well as water management correction is desirable [27, 28] if only with the help of the digital sensing. Actually, the soil-borne diseases, the water deficit and exceeding water salinity, in different way in the long run, may produce at canopy level, similar symptoms related to sap-flow deficiency, which could hardly be discriminated with a quick visive inspection, especially on large scale.

Precision farming principles require to connect as much information as possible derived from sensing systems to develop support tools for farmer’s decisions on the base of high throughput non destructive monitoring capability applicable on large cultivated surfaces [29–31].

The goal of this experiment was to early detect and identify, through the hyperspectral image analysis, the biotic (Fusarium wilt and Rhizoctonia rot) and abiotic (water and saline) stressors causing symptoms on wild rocket, to extend its application in the future to most of leafy vegetables. Direct measurements of water status, leaf colour and pigment contents were performed to ascertain the sensibility of wild rocket to such biotic and abiotic stress sources and to bear out the effectiveness of hyperspectral image technique.

## Materials and methods

### Plant pathogens

The phytopathogens used in this study were *R. solani* (AG-4) and *F. oxysporum f. sp. raphani* isolated from symptomatic cabbage and wild rocket plants, respectively. Both fungal strains are maintained on potato dextrose agar (PDA, Oxoid) slants at 20 °C and each isolate was preliminarily tested for pathogenicity on wild rocket cv Tricia. Pathogen inoculum preparation followed procedures described by Pane et al. [32, 33] to obtain *R. solani* infected millet and *F. oxysporum* conidial suspension, respectively.

### Plant material and experimental conditions

The *in planta* experiment was carried out in a glasshouse of the CREA-Research Centre of Vegetable and Ornamental Crops (Pontecagnano Faiano, Italy). Wild rocket (cv Tricia) seedlings were transplanted on 13 May 2019 in 20 cm plastic pots (five *per* each) filled with autoclaved soil-peat. Pots were arranged on bench and supplied manually with a basal NPK mix nutritive solution at three-day intervals. At 2 weeks after transplanting (28 May 2019), plants were subjected to five treatments during 1 week: plants watered to 100% water holding capacity (leaching 20% of the applied water and electrical conductivity (EC) of the irrigation solution of 1.53 dS m^−1^) (Control, C); infection by drenching 100 mL pot^−1^ of *F. oxysporum f. sp. raphani* strain conidial suspension (1.0 × 10^5^ conidia mL^−1^) (Fusarium stress, F); infection by amending 10 g pot^−1^ of *R. solani* colonized millet (Rhizoctonia stress, R); plants received 50% of water than the control (well-watered) plants (Water stress, W); plants irrigated at holding capacity, with EC of the irrigation solution of 3.7 dS m^−1^, after addition of NaCl (saline stress, S). This research was executed in a randomized complete block design with ten replications, and each replicate represented a pot with 5 plants.

In the greenhouse, climatic data were monitored: air temperature and relative humidity were registered and checked by the Agricontrol’s MCX Climate Control System. (Agricontrol Srl., Albenga, SV, ITALY) and radiation by a solar radiation sensor (model 6450, David instruments, Hayward, CA, USA, USA). The temperature ranged between 12 °C and 34 °C and the relative humidity between 29 and 90%. The maximum global radiation was 730 W m^−2^.

### Water status

The plant water status of rocket accessions was determined by the leaf relative water content (RWC_l_; %), the plant water potential (Ψ_pl_; MPa), the osmotic potential at full turgor (Ψ_100 s_; MPa), the electrolyte leakage (EL; %) and the dry matter content (DM; %). The RWC_l_ was measured in excised leaves harvested at midday (10:30–12:30 h solar time) using one leaf per plant of three plants per pot, providing an average pot RWC_l_, for five pots per treatment, according to the equation [34]:

1 $$RWC_{l} = \frac{FW - DW}{{TW - DW}} \times 100$$

Where FW, DW, and TW are the fresh, dry, and turgid weights (g), respectively, of the whole leaf.

Leaves were weighed immediately after collection to determine the fresh weight (FW). The cut end of each leaf was placed in distilled water and kept in dim light at 4 °C for 24–48 h till the turgid weight (TW) was reached and recorded. The dry weight (DW) was measured after air-drying the leaves at 70 °C for 48 h.

The determination of the Ψ_pl_ was estimated according to the method described by Scholander et al. [35], using a pressure chamber (Model 600 EXP Super Chamber, PMS Instrument Company, Albany, OR, USA) in five plants per treatment. The rocket plant was pulled out from the soil and the soil was carefully washed away from the roots, which were immediately submerged in a container of water and placed in the pressure chamber. The upper part of the plant was detopped with a razor blade, sealed in the chamber and pressurised. The pressure in the pressure chamber was raised using nitrogen gas at a rate of 0.02 MPa s^−1^ [36].

The Ψ_100s_ was measured in excised leaves harvested at midday (10:30–12:30 h solar time) using one leaf per plant in five plants per treatment. Leaves were placed by their petiole into flasks of distilled water and kept overnight in dim light at 4 °C to reach full saturation. After that, leaves were dried by filter paper to eliminate surface water, wrapped in aluminium foil and immediately frozen in liquid nitrogen (− 170 °C) and stored at − 30 °C. Before the measurements, samples were thawed and leaf sap was extracted for immediate determination of osmolality (mOsmol kg^−1^) using a freezing point osmometer (Osmomat 3000, Gonotec GmbH, Berlin, DE). The Ψ_100s_ in MPa was obtained by multiplying the osmolality with − 2.479 (conversion factor at 25 °C; [37, 38]).

Electrolyte leakage (EL) was determined as described by Lutts et al. [39]. Briefly, 10 pieces of leaves (10 × 10 mm) collected from four plants *per* plot were placed in individual vials containing 10 mL of distilled water. Samples were incubated at room temperature (25 °C) on a shaker (100 rpm) for 24 h. The initial electrical conductivity (EC_1_) of the bathing solution was measured using a conductivity meter (model Metrohm 6.0915.100, Metrohm Herisau, Switzerland). To measure total electrolytes released from leaf tissues, vials were then autoclaved at 120 °C for 20 min. The same samples were then autoclaved at 120 °C for 20 min and cooled at 25 °C to obtain the final electrical conductivity (EC_2_). The EL was calculated as:

2 $$EL\left( \% \right) = \frac{EC1}{{EC2}} \times 100$$

The dry matter content (DM) of the plant was expressed as weight percentage in 3 plants per pot and 5 pots per treatment and was calculated as dry weight (DW)/fresh weight (FW) × 100. In order to determine the DW, fresh plant material was dried in a thermo-ventilated oven at 70 °C until it reached a constant mass.

### Leaf color and pigments content

CIELAB (L*a*b*) leaf color coordinates were measured using a CR-210 Chroma Meter (Minolta Corp., Osaka, Japan) on leaf per plant of three plants per pot and ten pots per treatment. Measurements were done in duplicate, on mature and developed leaves of a similar age randomly chosen, on the two opposite lobes excluding the central rib and expressed as L*, a*, b* values. L* indicates lightness/darkness (0 = black, 100 = white), a* describes intensity in green − red (where a positive number indicates redness and a negative number indicates greenness), and b* describes the intensity in blue − yellow (where a positive number indicates yellowness and a negative number indicates blueness). Chroma (C) and hue angle (h) were estimated by the a* and b* values using the following equations:

3 $${\text{C }} = \left[ {\left( {a^{*} } \right) ^{2} + \left( {b^{*} } \right)} \right] ^{1/2}$$

4 $$h = \tan^{ - 1} \frac{{b^{*} }}{{a^{*} }}$$

Chroma indicates colour saturation, while hue is a measure of the angle in the CIELAB colour chart (0° or 360° indicates red hue, while angles below 270°, 180°, and 90° indicate blue, green, and yellow hue, respectively).

SPAD index was measured at the midpoint of one leaf per plant of three plants per pot and 20 pots per treatment, using a Minolta SPAD-502 chlorophyll meter (Konica-Minolta, Japan).

Chlorophylls (µg g^−1^ fresh weight) were extracted by homogenization of fresh leaf tissues (0.5 g) in acetone (80%). The resulting extracts were centrifuged at 4800 ×*g* for 15 min and the absorbance of solutions was measured at 662 and 647 for chlorophyll *a* and *b*, respectively, by a UV‐Vis spectrophotometer (model UV-1800, Shimadzu, Canby, US). Formula and extinction coefficients used for the determination of chlorophylls were described by Lichtenthaler and Wellburn [40]. The total chlorophyll content was calculated as the sum of chlorophyll *a* and *b*.

### Hyperspectral imaging

Hyperspectral images were immediately acquired, once rocket leaves were detached, using the SPECIM IQ camera (SPECIM, Spectral Imaging Ltd., Oulu, FI), working in the range of 400–1000 nm. A total of 204 wavelengths were considered along this range, with a probe spectral resolution of 7 nm. The camera carries a complementary metal–oxide–semiconductor (CMOS) sensor with a spatial sampling of 512 pixels and an image spatial resolution of 512 × 512 pixels [41]. Reflectance value was calculated automatically by the camera software. Two 46 W halogen lamps pointing at each corner of the object were used for stable lighting conditions of the scene. One image *per* replicate, containing 5 leaves, one for each treatment, was acquired, obtaining 30 images for a total of 150 leaves.

Samples were scanned by acquiring the entire surface of the leaf creating a hypercube dataset. Relative reflectance hyperspectral images were simultaneously computed by the camera software. White reference and dark frame and raw data were acquired during the measurement.

The equation for the computation of the Reflectance by the SPECIM IQ Camera is:

5 $${\text{Reflectance}} = { }\frac{{{\text{Raw}}\_{\text{data}}^{{{\text{t}}1}} - {\text{ Dark}}^{{{\text{t}}1}} }}{{{\text{Raw}}\_{\text{data}}^{{{\text{t}}2}} - {\text{ Dark}}^{{{\text{t}}2}} }}{ } \times { }\frac{{{\text{t}}2}}{{{\text{t}}1}}$$

The extraction of the spectral signature from each leaf was performed using the plugin of Quantum GIS software, called Point Sampling Tools that allowed a random sampling, from three regions of interest (ROIs) designed on the upper leaf surface on both sides and at the apex, of 10 pixel-point each, for a total of 30 spectral signature *per* leaf. The resulting dataset including 4500 spectra was submitted to the successive elaboration in “Artificial intelligence modelling” section.

Vegetation indeces were calculated by computing the spectral data according to the formulas reported in Additional file 1: Table S1.

### Statistical analyses

Differences among treatments for parameters of “Water status” sections and “Leaf color and pigments content” sections were analysed by one-way ANOVA test (at *P* ≤ 0.05 level) followed by a Duncan pairwise comparison test using Statgraphics Plus 5.1 (StatPoint Technologies Inc., Warrenton, VA, US).

### Artificial intelligence modelling

The hyperspectral images aforementioned, “Hyperspectral imaging” section, were analysed by means of an artificial intelligence approach aiming at classifying the spectra associated with the single pixel of the image. For each leaf, mean spectral values of the 204 wavelengths were calculated, and the dataset submitted for the artificial intelligence modelling was composed by 150 observations (*i.e.* 30 leaves for each class) × 204 wavelengths. To do this, a single layer feed forward artificial neural network (SLFN; [42]) was designed using a one-hidden layer architecture with 40 nodes and sigmoid activation functions and 5 SoftMax output neurons associated respectively with the following classes: control, Fusarium, Rhizoctonia, salinity stress and water stress. The artificial neural network (ANN) was trained with Scaled conjugate gradient backpropagation algorithm [43] as implemented in the deep learning MATLAB toolbox. The dataset was partitioned using 70% of the samples (105) as training set and 30 per cent of the data set as test set (45). The test set was used to validate the model. This partitioning was optimally chosen with the Euclidean distances calculated by the algorithm reported by Kennard and Stone [44], selecting parameters without a priori knowledge of a regression model. The cost function was minimized using the mean squared normalized error (MSE) error performance function with a 10^–10^ threshold on the gradient.

The confusion matrix of the percentages of pixel classification for each leaf was produced and an ANOVA test (H_0_: same mean) was performed to test the significance of differences among classified pixels into classes.

In order to understand which frequency values, among the 204 considered in the absorbance spectra, resulted to be more important in revealing the effects due to the presence of the different stresses, a variable impact analysis was also conducted. The variable impact $\Delta^{k}$ for the *k*-th frequency of the absorbance spectrum, was calculated in the following way. The complete data set (training set + test set) was considered made of *m* absorbance spectra. Each spectrum can be thought as a row vector **x** with *n* columns. The *m* spectra of the data set were stored in a matrix **X** having *m* rows and *n* columns. As a result, a generic element of that matrix is ${\text{X}}_{m}^{n}$. The spectra belong to 5 classes represented by a vector **y**. The output **y** is indeed a column vector with *m* rows obtained by applying the operator ***N*** to the matrix **X**:

6 $${\mathbf{y}} = {\varvec{N}}{\mathbf{X}}$$

In particular, y = ***N*X**_**k**_ is a row number representing the class of the k-th element of the data set and X_k_ is the row vector representing the k-th row of the **X** matrix (*i.e.*, the k-th spectrum of the data set). In this study ***N*** is a nonlinear operator and can be expressed as the tensor product of several linear and nonlinear operators. It represents, indeed, the converged SLFN. As a first step the first row X_1_ of the matrix **X** was considered and the operator ***N*** was applied *m* times to X_1,_ choosing each time a different value of ${\text{X}}_{1}^{1}$ among the values of X^1^, the latter being the first column of the matrix **X**. The 1-case dependent variable impact of the first variable $\Delta_{1}^{1}$ was then defined as:

7 $$\Delta_{1}^{1} = \mathop {\max }\limits_{{X_{1}^{1} \in X^{1} }} {\varvec{N}}{\text{X}}_{1} - \mathop {\min }\limits_{{X_{1}^{1} \in X^{1} }} {\varvec{N}}{\text{X}}_{1}$$

This procedure was repeated for all the *n* variables over all the data set. The *i*-case dependent variable impact of the *k*-variable $\Delta_{i}^{k}$ was then defined as:

8 $$\Delta_{i}^{k} = \mathop {\max }\limits_{{{\text{X}}_{i}^{k} \in {\text{X}}^{k} }} \left( {{\varvec{N}}{\text{X}}_{i} } \right) - \mathop {\min (}\limits_{{{\text{X}}_{i}^{k} \in {\text{X}}^{k} }} {\varvec{N}}{\text{X}}_{i} )$$

Finally, the variable impact of the *k*-th frequency of the spectra was then obtained by averaging $\Delta_{i}^{k}$ over all the *m* cases of the data set:

9 $$\Delta^{k} = \frac{{\mathop \sum \nolimits_{i = 1}^{m} \Delta_{i}^{k} }}{m}$$

The procedure described above is similar to that implemented in the Palisade software. The model was developed by using the MATLAB 9.7 R2019b Deep Learning Toolbox.

Once the network has been trained, the artificial intelligence model was applied on each hyperspectral image, classifying pixel by pixel each entire image. The result of this operation was quantitative (i.e., counting the pixels belonging to each class).

## Results

### Water status of plants

**Fig. 1:**
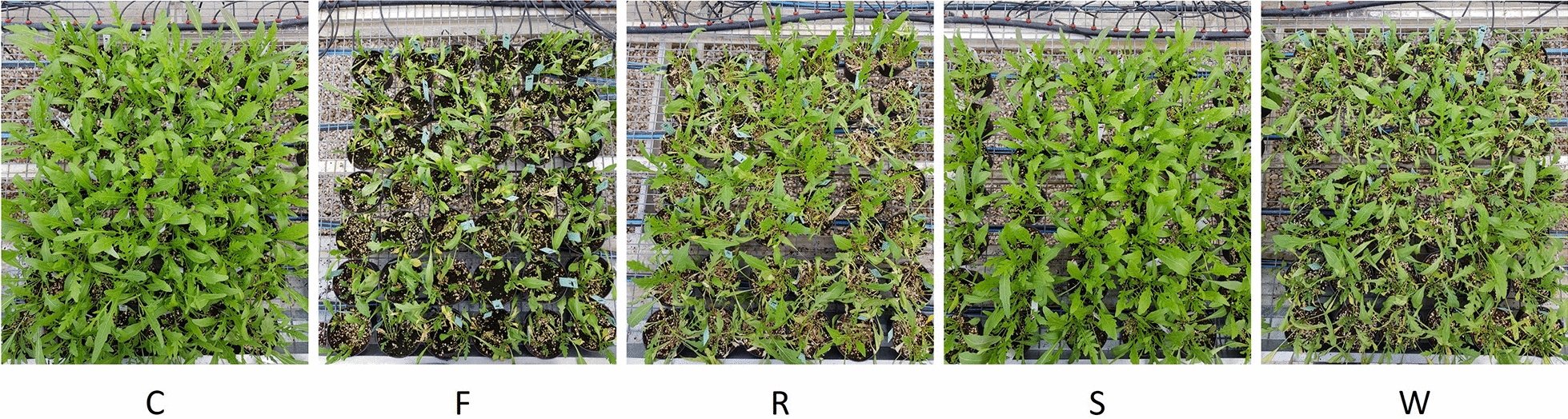
Wild rocket at the end of exposure period to stresses sources, *Fusarium oxysporum* f.sp. *raphani* (F), *Rhizoctonia solani* (R), salinity (S) and water deficit (W) compared to a healthy control (C)

Plant water status of wild rocket was determined at the end of the experimental period in the five treatments (Fig. 1). There were noticeable differences in water relations parameters, together with dry matter and SPAD index of wild rocket plants due to both abiotic and biotic stress sources applied (Table 1).

**Table 1:** Effects of the abiotic and biotic stresses sourced by *Fusarium oxysporum* f.sp. *raphani* (F), *Rhizoctonia solani* (R), salinity (S) and water deficit (W) compared to a healthy control (C), on the relative water content (RWC; %), plant water potential (Ψ_pl_; MPa), osmotic potential at full turgor (Ψ_100π_; MPa), electrolyte leakage (EL; %), dry matter (DM; %) and SPAD index (SPAD) in wild rocket seedlings at the end of the experimental period

	Treatments										*P*^a^
	C		F		R		S		W		
RWC (%)	89.29 ± 0.60	a^b^	83.60 ± 1.41	b	84.40 ± 2.24	b	78.51 ± 0.84	b	71.92 ± 2.66	c	***
Ψ_pl_ (MPa)	− 0.58 ± 0.03	a	− 0.69 ± 0.02	b	bdl^c^		− 0.72 ± 0.02	b	− 1.0 ± 0.04	c	***
Ψ_100π_ (MPa)	− 0.82 ± 0.02	b	− 0.68 ± 0.02	a	− 0.93 ± 0.04	c	− 0.84 ± 0.02	b	− 0.84 ± 0.03	b	***
EL (%)	52.19 ± 7.06	bc	83.22 ± 3.21	a	79.07 ± 6.84	a	47.88 ± 8.58	c	72.08 ± 10.94	ab	**
DM (%)	9.95 ± 0.54	b	11.15 ± 0.40	ab	13.81 ± 0.96	a	10.63 ± 0.23	b	15.43 ± 0.75	a	***
(SPAD)	50.85 ± 1.24	a	44.72 ± 1.67	b	45.01 ± 1.64	b	50.48 ± 1.40	a	47.26 ± 1.40	ab	**

^a^** and *** denote statistical significance at the 0.01 and 0.001 levels of significance, respectively.

^b^Different letters in the same line indicate significant differences among treatments, according to the Duncan’s test (*P* < 0.05).

^c^Ψ_pl_ was below detection level (bdl) under R treatment

RWC and Ψ_pl_ behaved in a similar way, showing C plants the highest values of these parameters (90% and − 0.58 MPa), while plants under water deficit showed the lowest RWC values (72%) and the most negative Ψ_pl_ (− 1 MPa). For plants exposed to the other treatments (F*,* R*,* and S) intermediate values were found. Ψ_pl_ was not measured in plants infected with *R. solani* due to its stem weakness didn’t allow its determination by the pressure chamber. The lowest values of Ψ_100s_ were obtained in plants infected with *R. solani*, (− 0.93 MPa) followed by abiotic stresses and control plants (on average − 0.83 MPa), while highest values were found in plants infected with *F. oxysporum* f. sp. *raphani* (− 0.68 MPa) (Table 1).

C and S treatment plants showed the lowest percentages of DM and EL (on average 50%) and the highest SPAD values (Table 1). The opposite happened for the biotic stressors and W treatments, which showed the highest EL percentages (on average 81% and 72% for biotic and W treatments, respectively) and the lowest SPAD values. The response of dry matter differed between biotic and W treatments, since plants under water stress showed the highest value of this parameter (15%), and in plants under biotic stressors this significant increase was only significative for plants infected with *R. solani* (≈ 14%) and not for those infected with *F. oxysporum* f. sp. *raphani* (11%) (Table 1).

**Fig. 2:**
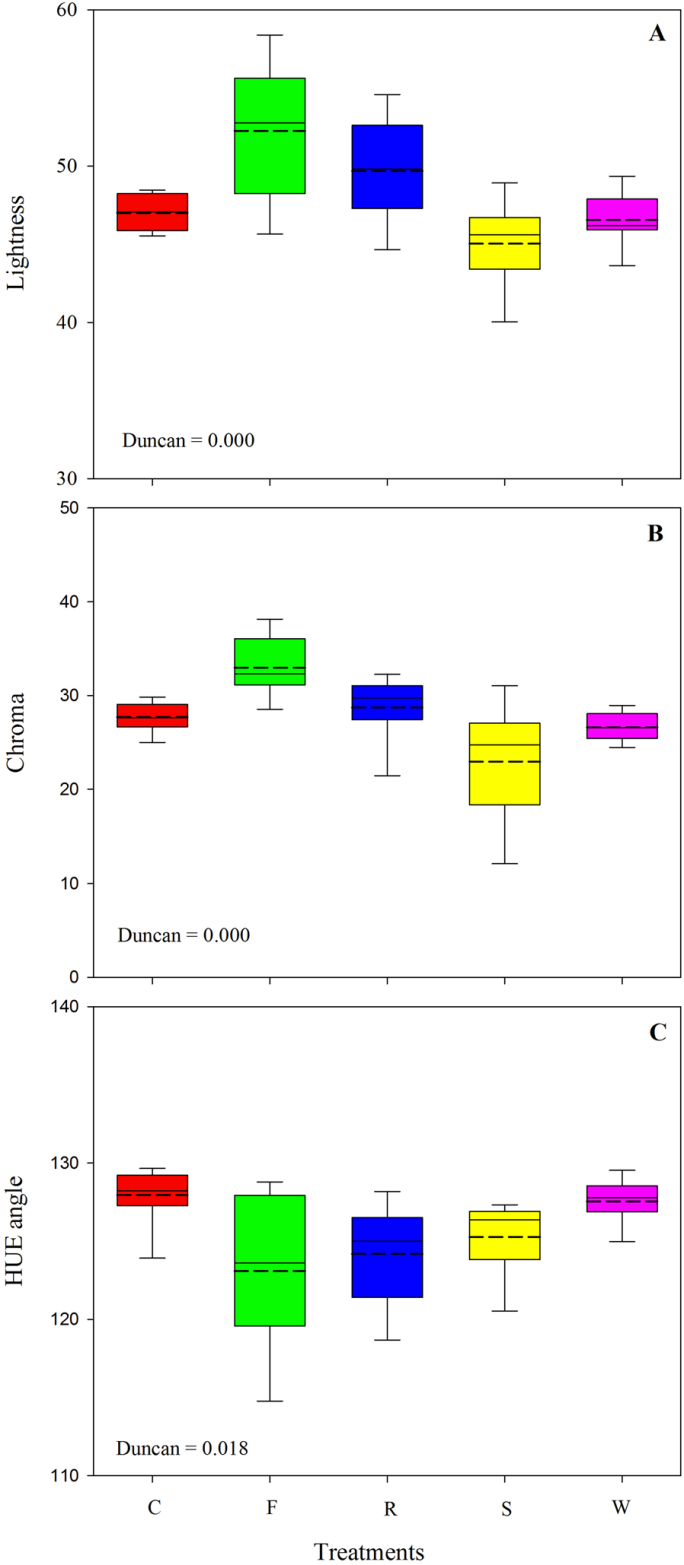
Colour parameters (Lighness; **A**, CHROMA; **B** and HUE; **C**) in wild rocket seedlings at the end of the exposure period to stresses sourced by *Fusarium oxysporum* f.sp. *raphani* (F), *Rhizoctonia solani* (R), salinity (S) and water deficit (W) compared to a healthy control (C). Boxplots are based on mean values of 30 repetitions. The solid line in the box indicates the median and the short dash line the mean. Boxplots show the 25 and 75% quantiles as the lower and upper limit of the box. The lower and upper whiskers represent the 5 and 95% percentile

In Fig. 2 the colour coordinates, lightness, chroma, and hue angle which characterized leaf colour are showed. Lightness was increased with biotic stresses, although more pronounced in plants infected with *F. oxysporum* f. sp. *raphani,* compared control and abiotic stressors ones (Fig. 2a). For Chroma, the highest values were found in plants with F treatment and the lowest with S one, the other treatments showing intermediate values (Fig. 2b). Hue angle decreased with biotic stresses in comparison with abiotic stresses and C treatments, and these values were statistically equal for the two fungal infections (Fig. 2c).

**Fig. 3:**
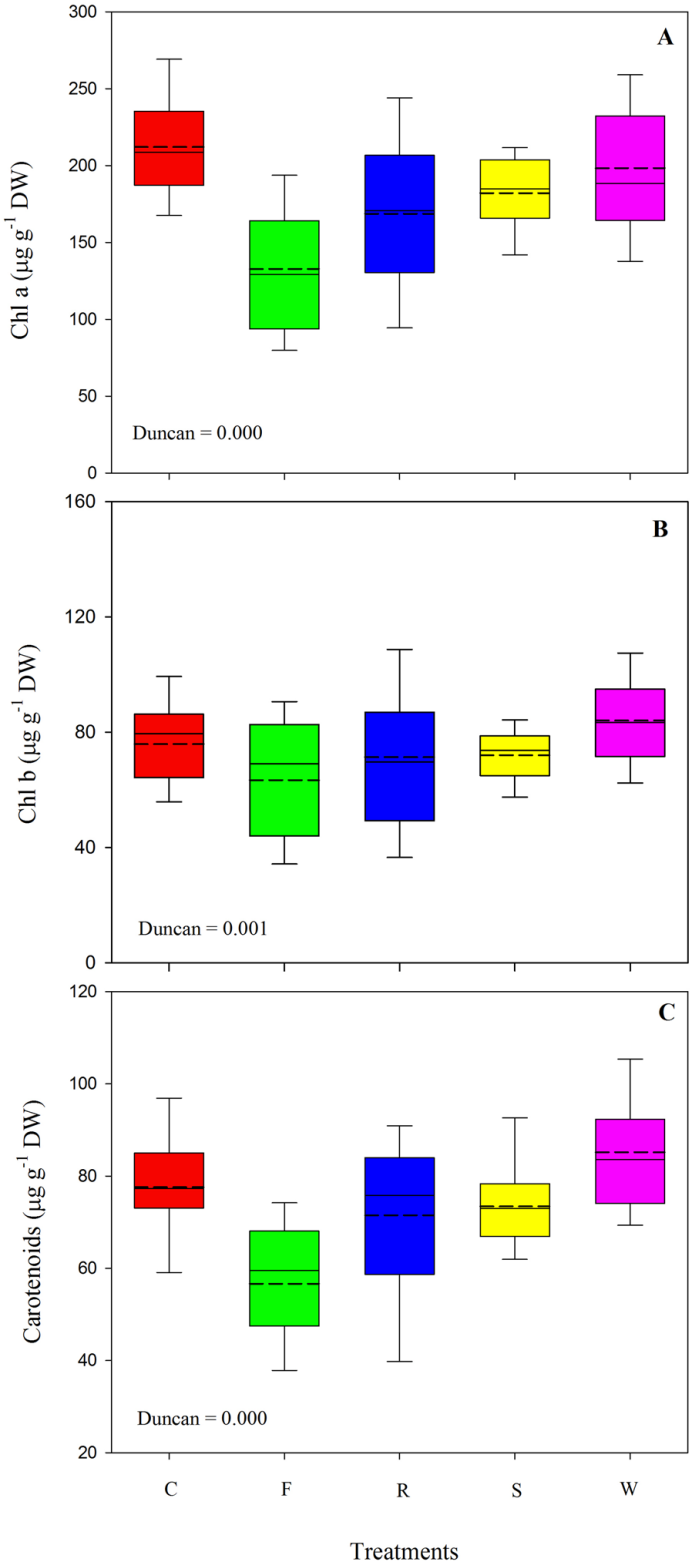
Chlorophyll and carotenoids content (Chl a; **A** Chl b; **B**, and carotenoids; **C**) in wild rocket at the end of the exposure period to stresses sourced by *Fusarium oxysporum* f.sp. *raphani* (F), *Rhizoctonia solani* (R), salinity (S) and water deficit (W) compared to a healthy control (C). Boxplots are based on mean values of 30 repetitions. The solid line in the box indicates the median and the short dash line the mean. Boxplots show the 25 and 75% quantiles as the lower and upper limit of the box. The lower and upper whiskers represent the 5 and 95% percentile

Chlorophyll *a, b* and carotenoids content showed a significant decrease only in plants infected with Fusarium compared to the other treatments, where values of these parameters were statistically equal (Fig. 3a, b, c). Reductions were on average of 60, 15, and 25μg g^−1^ for chlorophyll *a, b* and carotenoids, respectively.

### VIS–NIR reflectance patterns and vegetation indices

**Fig. 4:**
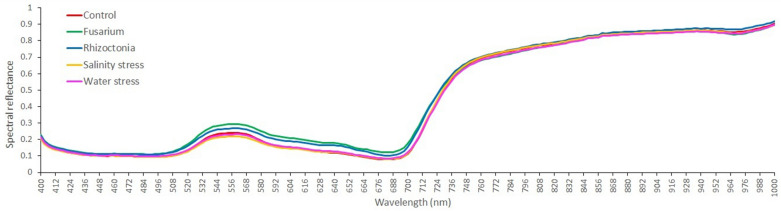
Hyperspectral reflectance signature in VIS–NIR spectral region of leaves from wild rocket collected 1 week after the exposure period to stresses sourced by *Fusarium oxysporum* f.sp. *raphani* (F, green), *Rhizoctonia solani* (R, blue), salinity (S, yellow) and water deficit (W, pink) compared with healthy control (C, red)

Means of all pixel-wise spectral data from stressed and reference samples is showed in Fig. 4, where an increased reflectance is highlighted around the photosynthetically active wavelengths in the VIS region for the Fusarium and Rhizoctonia samples compared to the other treatments. Forty-nine out of 54 hyperspectral vegetation indices considered in this study provided significant (*P* ≤ 0.05) differences among treatments, in at least one case (Additional file 1: Table S2). Twenty-four indices distributed along the various target categories of plant vitality and vegetation, pigment content and photochemical activity, were able to differentiate samples based on treatments. Interestingly, Modified Soil Adjusted Vegetation Index (mSAVI), Simple Ratio R_550_-to-R_670_ (G), Lichtenthaler indices 3 (LIC3), Ratio Analysis of Reflectance Spectra (RARS), Normalized Difference Vegetation Index (NDVI) and Green NDVI (gNDVI) displayed a treatment rank ordering in agreement with physiological findings: Fusarium positioned at the opposite end of the control. Water index (WI) was the lowest under Rhizoctonia, whereas DVI, Soil Adjustment Vegetation index (SAVI), Transformed Soil Adjustment Vegetation index (TSAVI) and Red-Edge Position Linear Interpolation were it for Fusarium. Simple Ratio Pigment Index and Modified Chlorophyll Absorption in Reflectance Index were lower for S and W stresses, respectively, compared to the other treatments.

### Artificial intelligence modelling based on VIS–NIR spectral data

**Fig. 5:**
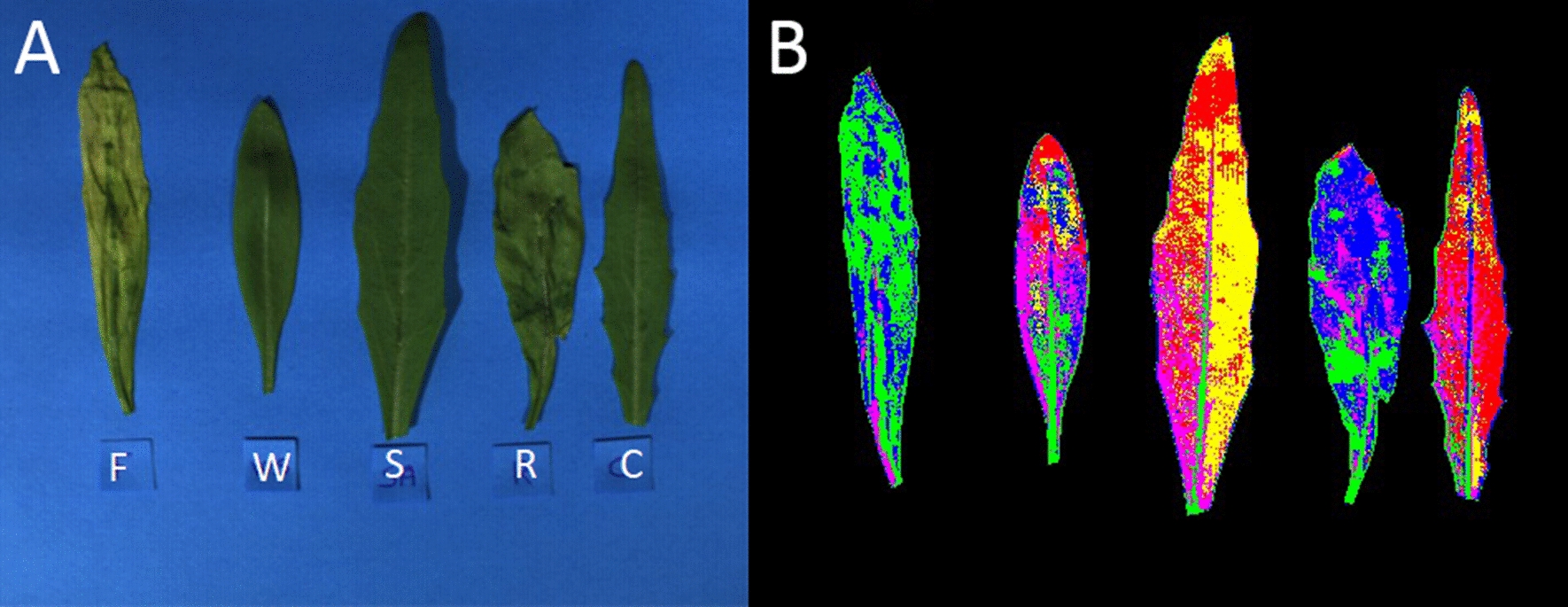
Example of single pixel classification. Original image (in RGB; **A**) and classified image (**B**) through the trained ANN model used as a pixel classifier on the multispectral images of leaves collected 1 week after the treatment application. In B the colors refer to the different classes, i.e. wild rocket exposed to stresses sourced by *Fusarium oxysporum* f.sp. *raphani* (F, green), *Rhizoctonia solani* (R, blue), salinity (S, yellow) and water deficit (W, pink) compared with healthy control (C, red)

The ANN trained model has a hidden layer size of 40 nodes and the algorithm converged after 955 iterations (3 s). Table 2 reports the characteristics and principal results of the ANN model used to predict the stress from the 150 (VIS–NIR) spectral data. All the 105 spectra in the training set were correctly classified. In testing, there were 12 misclassified spectra. The most misclassified stress was W (5 misclassified samples) followed by F (3 misclassified samples), R and S (2 misclassified samples). This was probably due to the spectral modification associated with the biological consequences induced by the 4 stresses (stoma enclosure and temperature enhancement). The trained ANN model was used as a pixel classifier on the multispectral images shown in Fig. 5 where different colors refer to the different classes.

**Table 2:** Characteristics and principal results (number of cases, training time, number of trials, percentage of bad predictions) of the SLFN model (training and internal test) in predicting the classification of the different treatments: control, Fusarium, Rhizoctonia, salinity and water deficit

Training (70%)	
Number of cases	105
Number of hidden layers	1
Number of nodes	40
Training time	00:00:03
Number of trials	955
% bad predictions	0.0
Testing (30%)	
Number of cases	45
% bad predictions (N)	26.7 (12)

The confusion matrix of the percentages of pixel classification for each leaf is reported in Table 3. The results of the ANOVA test (H_0_: same mean) showed how percentages of correctly classified pixels (positioned on the main diagonal of the Table 3) are always significantly higher than those wrongly classified.

**Table 3:** Confusion matrix of the percentages of pixel classification for each leaf class, i.e. stresses sourced by *Fusarium oxysporum* f.sp. *raphani* (F), *Rhizoctonia solani* (R), salinity (S) and water deficit (W) compared to control (C) treatment

Treatments	Output				
	C	F	R	S	W
C	0.34 ± 0.10a	0.14 ± 0.07bc	0.12 ± 0.06c	0.19 ± 0.09b	0.21 ± 0.10b
F	0.05 ± 0.06c	0.56 ± 0.18a	0.16 ± 0.10b	0.06 ± 0.08c	0.18 ± 0.09b
R	0.08 ± 0.11c	0.22 ± 0.13b	0.45 ± 0.19a	0.08 ± 0.09c	0.18 ± 0.11b
S	0.16 ± 0.11bc	0.15 ± 0.08c	0.14 ± 0.08c	0.34 ± 0.08a	0.20 ± 0.09b
W	0.10 ± 0.07c	0.21 ± 0.14b	0.20 ± 0.13b	0.12 ± 0.09c	0.37 ± 0.12a

ANOVA test (H_0_: same mean) results are reported using letters. Horizontally reading, equal letters correspond to no significant differences

**Fig. 6:**
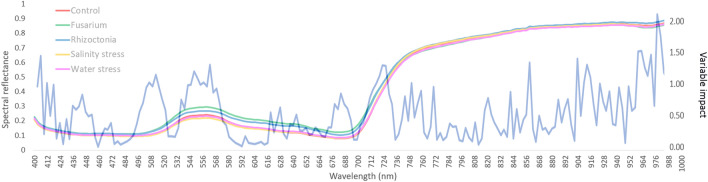
Mean VIS–NIR spectral data (left side axis) for wild rocket at the end of the exposure period to stresses sourced by *Fusarium oxysporum* f.sp. *raphani* (F, green), *Rhizoctonia solani* (R, blue), salinity (S, yellow) and water deficit (W, pink) compared with healthy control (C, red). Variable impact (right side axis) evidenced with dark blue line

Observing the variable impact of spectral values, the most informative ones ranged within the following frequencies: 492–504 nm, 540–568 nm, 712–720 nm, 855 nm, 900–908 nm and 970 nm (Fig. 6)

## Discussion

Wild rocket baby-leaf is currently grown in very intensive greenhouses than are essentially conditioned by relative humidity, fertigation, coastal soil and climatic conditions, high seeding density, and continuous recultivation in monoculture, which potentially expose the crop to various biotic and/or abiotic stressors [45, 46]. The success of management strategies aimed at ensuring the best growth settings to achieve expected yields and earns, relies on early identification and targeted control of etiological factors. Hyperspectral imaging has been proposed, in recent years, for the rapid, non-destructive and object-oriented classification of plant physiological changes induced by the harmful pathogens pressure and/or adverse environmental conditions to plant growth [47]. Digital monitoring of the plant stress onset can improve the effectiveness of control methods by supporting farmer decisions and quickly advising precise intervention.

The current study focused on deficiencies in wild rocket caused by two soil-borne pathogens, *F. oxysporum* f. sp. *raphani* and *R. solani*, and two sources of abiotic stresses attributed to water deficit and salinity. Related aboveground symptoms may not be properly distinguishable by visual detection, and, especially in the early stages of their evolution, may be confused with each other, delaying the application of the most appropriate remedies. The root system is the main target of salt and drought in the soil and can modulate physiological responses in the aerial part of the plant, resulting in non-specific symptoms as a result of the nutrient flux and water relations involved [48].

In the current study, water relations of wild rocket plants, were mainly affected by abiotic stress, as shown by decreases in RWC and Ψ_pl_, but with a greater impact for water deficit, where higher EL and DM were also found. Rates of passive ion leakage from stress damaged plant tissues are used as a measure of alterations in membrane permeability, and to characterise plant cell membrane stability [49], suggesting that plant cell membranes were damaged under water deficit in this study. The higher dry matter content of wild rocket plants under water deficit conditions suggests an improvement of their shoot dry weight. Instead, plants under salinity conditions showed the same EL and DM values as control plants, and lower RWC and Ψ_pl_ values than control plants but statistically higher than those of the water-stress treatment, due on the one hand to short-term exposure to sodium chloride and on the other hand to the known tolerance of rocket plants to salinity. Barbieri et al. [50], established an EC of 5 dS m^−1^ in the nutrient solution for an improvement in dry matter content, visual appearance, carotenoids and phenols of *E. sativa*, while Bonasia et al. [51] give an EC threshold of 3.5 dS m^−1^ in the nutrient solution for growing wild rocket, which enhances leaf texture, visual quality, and antioxidant compounds, and reduces nitrate content, without a decrease in dry weight. Indeed, saline stress has been applied by other authors [52, 53] to improve vegetable quality by increasing the production of secondary metabolites and sensory characteristics and reducing anti-nutritional factors.

At the beginning of the Rhizoctonia basal rotting and Fusarium wilting, wild rocket plants decreased their RWC and Ψ_pl,_ although it was not possible to determine the values of the latter parameter in the leaves of diseased plants by Rhizoctonia, as it happened under abiotic stresses. However, the Ψ_100s_ highlights a variable response among biotic factors, likely related to the specificity of stressors mechanisms in damaging plant. The lower values of Ψ_100s_ found in plants infected with *R. solani,* compared to other treatments, are indicative of osmotic adjustment, which involves the net accumulation of solutes in a cell in response to stress. Pérez-Pérez et al. [54] stated that, consequently, the osmotic potential decreases, which in turn attracts water into the cell and allows turgor to be maintained, although it was not possible to know the extent of the plant stress as it was not possible to measure Ψ_pl_ in plants infected with *R. solani*. *F. oxysporum f. sp. raphani* enters the host through the root and then develops endophytically to invade the xylem vessels, not externally as happens with *R. solani*. Plants under Fusarium infection use as defence reaction the production of physical barriers (i.e. gums) to block the progression of the pathogen. Nevertheless, the occlusion of the vessels prevents the mycelium spreading, but also drastically reduces the entry of solutes and water from the root medium. As consequence, their Ψ_100s_ did not decrease, nay values were even higher than in the control plants, suggesting a decrease in turgor potential. Ψ_pl_ reflects the symptoms of a water stress in the plant, but the relative contribution of the two main components, osmotic and turgor potential to leaf water potential can experience significant differences depending on the species and/or treatment (stress) applied. Increased resistance of water flow from the substrate to the plant has been observed in several species, especially under water stress conditions [55–57] and, in our case this phenomenon may have reduced water transport to the leaves due to the gradual closure of the xylem vessels by the Fusarium mycelium. Furthermore, both fungi strongly increased electrolyte leakage suggesting that stress-induced injury of cell membrane due to oxidative damage, could be related to the turgor loss.

The values of colour space coordinate suggested that the leaves of plants under biotic stresses were more yellowish green (decreased in hue angle), lighter (increased in lightness) and gained in saturation (increased in chroma) than the leaves of the control plants and those of the abiotic stresses. This indicates that leaf colour is modified by biotic stresses, and that, the discoloration resulting from both pathogens could be due to chlorophyll breakdown as also suggested by the lower SPAD values. However, spectrophotometric pigment evaluations revealed a significant reduction in the chlorophyll *a* and *b*, and carotenoids content only in Fusarium diseased plants compared to the other treatments. As it has been demonstrated on tomato, decline in the xylem flux due to Fusarium wilting is very detrimental to the photosynthetic system deprived of active pigments as early as 6–8 days after infection is started [58]. In contrast, *R. solani* did not affect chlorophyll concentration, as it was previously observed on Chinese cabbage over a comparable period [59].

Some vegetation indices, calculated on the bases of reflectance data targeted on plant vitality and chlorophyll content (NDVI and G), healthiness (mSAVI), and carotenoid content (LIC3 and RARS), agreed with the patterns of variations observed for the physiological parameters. Vegetation indices have already been proposed as a reliable method to classify plant diseases and stresses by synthetizing hyperspectral outputs for the purpose of early identification [60]. They elaborate in narrower spectrum data ranges that carry biological meanings. In the current study an artificial intelligence model based on hyperspectral reflectance of leaves was developed for the first time, achieving very good performance (only 26.7% of bad predictions). The dataset was further mined, and promising insights were obtained to perform an accurate classification of the source of the symptoms. ANN modelling using a pixel classifier was able to separate wild rocket treatments on the basis of the significant differences occurring in the leaf hyperspectral signatures by assigning higher discriminant weight to narrow wavelength ranges 492–504 nm, 540–568 nm and 712–720 nm, which fall within the VIS blue, green and red ranges and to 855 nm, 900–908 nm and 970 nm of the NIR spectrum. These ANN filtered reflectance regions, which were previously identified to refer about plant stresses, as they were related to leaf pigments and changes in cell structures. Narrowed blue and red ranges were previously found to be indicative of shifts in leaf pigment absorption [61] and chlorophyll content [62] due to ongoing plant stresses. Recently, a trained Random Forest model was applied to explore non-redundant bands falling in the violet-blue light region that can classify powdery mildew-affected wild rocket leaves [63]. Reflectance wavebands within green pigment indices selected from a wavelet-based optimal regression model as a predictor of active chlorophyll quantification [64]. Gitelson et al. [65] exploiting the high sensitivity of the VIS green channel to chlorophyll *a* concentration adapted the green-NDVI index to monitor photosynthetic activity and related plant stresses. Indeed, in this study, the gNDVI values proved to be gradated on the stress magnitude of the treatments as well as the G index, calculated as R_550_-to-R_670_ simple ratio, LIC3 (R_440_/R_730_) and RARS (R_746_/R_513_). Instead, the Photochemical Reflectance Index (PRI), calculated on R_531_ and R_570_, clearly separated stressed and non-stressed samples at very early stages of the present experimental conditions. The PRI was proposed by Meroni et al. [66] for early remote detection of incipient ozone stress on white clover, while on barley, it detected drought stress at 8 days after the complete water deprivation [67].

On the other hand, reflectance in the 950–970 nm region was found to be indicative of plant water status in gerbera, while, on the same crop, the R_970_-to-R_900_ ratio closely followed shifts in relative water content [68]. As matter of fact, PRI and WI were found to be among the most sensitive hyperspectral indices for assessing the water status of tomato under different irrigation regimes [69] and wheat under genetic selection for drought resistance [70].

Similar findings were previously observed on sugar beet, where nematode-induced posterior drought and Rhizoctonia wilting were significantly classified by canopy imaging with the carotenoids/chlorophyll a dependent Structural Independent Pigment Index, Simple Ratio Pigment Index, and WI indices [71]. Analogously, Susič et al. [72] used a partial least square-support vector machine approach to individuate wavebands in the highly discriminatory shortwave infrared spectral regions of the tomato canopy response to root-knot nematodes and soil water deficiency. In this regard, the experimental pipeline capable of classifying stresses as early as 12 days after initiation was described by Žibrat et al. [73]. Applying a linear regression analysis, Manganiello et al. [74] recently found the interactive combination of hyperspectral vegetation indices TSAVI + SAVI and Triangular Vegetation Index able to predict baby-leaf infection levels of three different soil-borne pathogens, including *R. solani* on wild rocket, as modulated by treatments with biocontrol agents *Trichoderma* spp.

## Conclusion

This study provided significative spectral information related to the evaluated stresses as corroborated by the physiological measurements. Fusarium wilting strongly affected water relations in the infected wild rocket plants, which showed the poorest cell integrity, chlorophyll content and greenness. Rhizoctonia disease proved to be the second most impactful stress under the experimental conditions as a result of root and collar rot, while abiotic stresses, especially salinity barely showed the effects induced by the water stress in the substrate. The reflectance-based findings of the present work constitute a valuable collection of data on the identification of putative bands that can be used for monitoring multiple stresses in wild rocket, to be employed prospectively as an input light-filter of cheaper and more feasible devices for precision stress assessment in this species, and extendable to other leafy vegetables. The potential of hyperspectral technology for innovative and high-performing detection tools is here enhanced by a model based on artificial neural network that allowed the identification of narrow spectral regions and single wavelengths highly sensitive to early shifts in reflectance profiles of stressed wild rocket due to changes in photosynthetically active pigments (VIS: 492–504, 540–568 and 712–720 nm) and water status (NIR: 855, 900–908 and 970 nm). This information can be used in the field of technological innovation for the design of new optoelectronic probes to support farmers in their choices, e.g. guiding both phytosanitary and irrigation treatments through machine vision.

## Supplementary Information

**Additional file 1: Table S1.** Indeces used for comparison of treatments in this work. **Table S2.** Effects of the abiotic and biotic stress on the vegetation indeces at the end of the experimental period.

## Data Availability

The datasets and field sampling data used in this study are available from the corresponding author on reasonable request.
