# Supplementary material for: Sorting biotic and abiotic stresses on wild rocket by leaf-image hyperspectral data mining with an artificial intelligence model
Source: Plant Methods. 2022 Apr 2;18:45. doi: 10.1186/s13007-022-00880-4 (PMC8977030; doi:10.1186/s13007-022-00880-4)
Supplement: Supplementary file 1 — Additional file 1: Table S1. Indeces used for comparison of treatments in this work. Table S2. Effects of the abiotic and biotic stress on the vegetation indeces at the end of the experimental period. [file 13007_2022_880_MOESM1_ESM.docx]

**Table S1.** Indexes used for comparison of treatments in this work.

| **Acronym** | **Index** | **Formula** | **Description** | **Reference** |
| --- | --- | --- | --- | --- |
| **NDVI** | Normalized Difference Vegetation Index (NDVI) | (R670-R800)/(R670 + R800) | plant vitality and Chlorophyll content | Rouse et al., 1973 |
| **PRI** | Photochemical reflectance index (PRI) | (R531-R570)/ (R531+ R570) | photochemical activity | Gamon et al. 1992. |
| **PSSRa** | Pigment-specific simple ratio (PSSR) | R800/R680 | Chlorophyll a | Blackburn, et al., 1998 |
| **PSSRb** | Pigment-specific simple ratio (PSSR) | R800/R635 | Chlorophyll b |  |
| **PSSRc** | Pigment-specific simple ratio (PSSR) | R800/R470 | carotenoid |  |
| **WI** | Water Index | R900/R970 | Water content | Peñuelas et al. 1997 |
| **RE-NDVI** | Red Edge NDVI | R750 - R705 / R750 + R705 | Vegetation |  |
| **SRI** |  | R800/ R 680 |  |  |
| **PSRI** | Plant Senescence Reflectance Index |  |  | [Merzlyak et al. 1999](https://www.indexdatabase.de/db/r-single.php?id=511) |
| **NPQI** | Normalized Difference 415/435 Normalized Phaeophytinization Index , Normalized difference pigment index NDPI | R415-R435/ R415+R435 | Vegetation, chlorophyll, stress | [Barnes, J. et al. 1992](https://www.indexdatabase.de/db/r-single.php?id=6) |
| **SIPI** | Structure Intensive Pigment Index 1 | R800 - R445 / R800/ R680 | Vegetation, Chlorophyll | [Penuelas J., et al.1995](https://www.indexdatabase.de/db/r-single.php?id=703) |
| **LRDSI** | Leaf rust disease severity index (LRDSI) | 6.9 × (R605/R455) − 1.2 |  | Ashourloo D. et al. 2014 |
| **RDVI** | Renormalized Difference Vegetation Index | (R800− R670) / (R800 + R 670)^0.5 | plant vitality and Chlorophyll content | Roujean et al., 1995 |
| **OSAVI** | Optimized Soil Adjusted Vegetation Index | (1+0.16) x (R800 − R 670) / (R 800 + R670+ (0.16)) | sparse vegetation where soil is visible through the canopy | Rondeaux et al., 1996 |
| **MCARI1** | Modified Chlorophyll Absorption in Reflectance Index 1 | 1.2 x [2.5 × (R800− R670)− 1.3 × ( R 800− R 550)] | plant vitality and chlorophyll content | Haboudane et al. 2004 |
| **MSAVI** | Modified Soil Adjusted Vegetation Index hyper | 0.5 x [2 x R 800 + 1 − √(2 x R 800 + 1 ) x 2 − 8 x (R800 − R670)] | healthy vegetation. | Qi et al., 1994 |
| **TCARI** | Transformed Chlorophyll Absorbtion Ratio | 3 x [(R700−R670) − 0.2 x (R700 − R550) x R700/ R670)] | plant vitality and Chlorophyll content | Haboudane et al., 2002 |
| **TCARI/OSAVI** |  | Combined TCARI & OSAVI | plant vitality and Chlorophyll content | Haboudane et al., 2002 |
| **TVI** | Triangular Vegetation Index | 0.5 x [120 x (R750 − R550) − 200 x (R670 − R550)] | plant vitality and Chlorophyll content | Broge et al., 2001 |
| **G** | Simple Ratio 550/670 Greenness Index | R550 / R670 | plant vitality and Chlorophyll content | Smith et al., |
| **ZTM** | Zarco - Tejada - Miller Index | R750/ R710 | Chlorophyll content estimation | Zarco-Tejada et al., 2001 |
| **VOG1** | Simple Ratio 740/720 | R740/R720 | plant vitality and Chlorophyll content | Vogelmann et al.,1993 |
| **CAR** | Simple ratio 515/570 | R515/R570 | Carotenoids content | Zarco-Tejada et al., 2013 |
| **LIC3** | Simple Ratio 440/740 Lichtenthaler indices 3 | R440/R740 | Carotenoids content | Lichtenthaleret al., 1996 |
| **RARS** | Simple Ratio 760/500 Ratio Analysis of Reflectance Spectra | R746/R513 | Carotenoids | Chappelle et al., 1992 |
| **PRI515** | Normalized Difference 515/531 Photochemical Reflectance Index 515/531 | R515 − R531 / R515 + R531 | Carotenoids | Hernández-Clemente et al., 2011 |
| **PRIn** |  | PRI / RDVI * (R700/R670) | Carotenoids | Zarco-Tejada et al., 2013 |
| **ARI** | Anthocyanin Reflectance Index (ARI) | (1/550) - (1/700) | Sensitive to changes in carotenoid pigments | Gitelson et al., 2001 |
| **RVSI** | Red-Edge Stress Vegetation Index | R800 − R670 / (R800 + R670) 0.5 | plant vitality | Roujean et al., 1995 |
| **HVI** | Hyperspectral Vegetation Index | R743 / R692 | plant vitality | Gitelson et al. 1996 |
| **GI** | Greeness index | R539 / R682 | plant vitality | Zarco-Tejada et al. 2005 |
| **DVI** |  | R782 − R675 | plant vitality |  |
| **RVI** | Ratio vegetation index (RVI) | R493/R678 | plant vitality | Tilley et al., 2003 |
| **SAVI** | Soil Adjustment Vegetation Index | ((R782-R675)/(R782 + R675 + 0.2)) (1.2) | Crop Parameters | Baret and Guyot, |
| **TSAVI** |  | R782 − R675 | Crop Parameters | Baret and Guyot, |
| **PVI** | Perpendicular Vegetation Index | (R800 − 0.2R670 − 0.6)/1.019 |  |  |
| **NDVI 705** |  | (R750 − R705)/(R750 + R705) | Vegetation | Gitelson et al. |
| **mNDVI705** | modified NDVI | (R750 − R705)/(R750 + R705 − 2 R445) | Vegetation | Huete et al., |
| **mSR705** | Modified Simple Ratio 705 | (R750 − R445)/(R705 + R445) | Vegetation | Wu et al., 2008 |
| **gNDVI** | Green NDVI | (R750 − R550)/(R750 + R550) | Vegetation | Buschmann, et al. 1993 |
| **VOG2** | Simple Ratio 734/747 Vogelmann indices | (R734 − R747)/(R715 + R726) | plant vitality and Chlorophyll content | Vogelmann et al., 1993 |
| **VOG3** | Simple Ratio 734/747 Vogelmann indices 3 | (R734 − R747)/(R715 + R720) | plant vitality and Chlorophyll content | Vogelmann et al, 1993 |
| **HNDVI** |  |  |  |  |
| **MCARI** | Modified Chlorophyll Absorption in Reflectance Index | R712 ×(R712−R682)−0.2(R712− R539)] / R682 | Chlorophyll absorption | Daughtry et al., 2000 |
| **WBI** | Water band index(WBI) | R950/R900 |  | Xu et al., 2007 |
| **FWBI1** | Floating-position water band index (FWBI1) | R900/Min [R930−R980] |  | Strachan et al., 2002 |
| **FWBI2** | Floating-position water band index (FWBI2) | R920/Min [R960 to R1000] |  | Harris et al., 2006 |
| **VARIg** | Visible atmospherically resistant index green | (RGreen-RRed)/(RGreen+RRed−RBlue) | estimate the fraction of vegetation | Gitelson et al., 2002 |
| **SRPI** | Simple Ratio Pigment Index (SRPI) | R430/R680 | Vegetation / Chlorophyll | Penuelas et al., 1994 |
| **Lomin** | Minimum band reflectance ranging from 640 to 680 nm | Min [R640 to R680] |  | Chen et al., 2011 |
| **RGRcn** | Red green ratio chlorophyll content (RGRcn) | (R612+R660)/(R510+R560) | Chlorophyll content | Steddom et al., 2003 |
| **AI** | Anthocyanin index (AI) | (R600−R699)/(R500−R599) |  | Gamon and Surfus, 1999 |
|  | **R705/(R717+R491)** | R705/(R717+R491) |  | Tian et al ., 2011 |
| **REP** | Red-Edge Position Linear Interpolation | R700 + 40[(R670 + R780)/2 − R700]/(R740 − R700) | Hyperspectral remote sensing - Red-edge position | Guyot et al. 1988 |

**Table S2.** Effects of the abiotic and biotic stress on the vegetation indexes at the end of the experimental period.

| **Index** | **C** | | **F** | | **R** | | **S** | | **W** | |
| --- | --- | --- | --- | --- | --- | --- | --- | --- | --- | --- |
| **NDVI** | 0.8129±0.0021 | a | 0.7406±0.0232 | c | 0.7664±0.0099 | bc | 0.8100±0.0022 | ab | 0.7980±0.0036 | ab |
| **PRI** | -0.0461±0.0021 | b | -0.0346±0.0052 | ab | -0.0316±0.0035 | a | -0.0423±0.0022 | ab | -0.0376±0.0019 | ab |
| **PSSRa** | 9.4812±0.1174 | a | 7.7712±0.3987 | b | 7.7913±0.2791 | b | 9.3297±0.1206 | a | 8.7095±0.1553 | ab |
| **PSSRb** | 6.6091±0.0928 | a | 5.3396±0.2914 | b | 5.2182±0.2076 | b | 6.4682±0.0925 | a | 6.1774±0.1263 | a |
| **PSSRc** | 0.8995±0.0034 | ab | 0.8997±0.0040 | ab | 0.8912±0.0059 | b | 0.9097±0.0041 | a | 0.8942±0.0034 | ab |
| **WI** | 0.9933±0.0027 | ab | 1.0032±0.0034 | a | 0.9869±0.0053 | b | 1.0025±0.0039 | a | 0.9943±0.0033 | ab |
| **Red Edge NDVI** | 0.3976±0.0040 | a | 0.3102±0.0165 | b | 0.3329±0.0107 | b | 0.3872±0.0038 | a | 0.3911±0.0048 | a |
| **SRI** | 9.7277±0.1207 | a | 8.0260±0.4071 | b | 8.0608±0.2830 | b | 9.5755±0.1251 | a | 8.9649±0.1582 | ab |
| **PSRI** | -0.0352±0.0011 | b | -0.0109±0.0055 | ab | 0.0010±0.0105 | a | 0.0017±0.0080 | a | -0.0179±0.0035 | ab |
| **NPQI** | 0.1069±0.0030 | a | 0.1067±0.0035 | a | 0.1025±0.0027 | a | 0.1030±0.0019 | a | 0.1091±0.0027 | a |
| **SIPI** | 0.7269±0.0079 | a | 0.7418±0.0107 | a | 0.7452±0.0100 | a | 0.7314±0.0081 | a | 0.7162±0.0085 | a |
| **LRDSI** | 8.0637±0.1255 | b | 9.7529±0.4321 | a | 9.1713±0.2427 | a | 7.8490±0.1808 | b | 7.9251±0.1282 | b |
| **RDVI** | 0.7491±0.0034 | a | 0.6860±0.0193 | b | 0.7176±0.0080 | ab | 0.7474±0.0033 | a | 0.7329±0.0048 | a |
| **OSAVI** | 0.7925±0.0021 | a | 0.7228±0.0219 | b | 0.7504±0.0091 | ab | 0.7896±0.0018 | a | 0.7765±0.0038 | a |
| **MCARI1** | 1.2476±0.0118 | a | 1.1873±0.0299 | a | 1.2263±0.0164 | a | 1.2106±0.0159 | a | 1.2014±0.0144 | a |
| **MSAVI** | 0.7865±0.0025 | a | 0.7168±0.0213 | c | 0.7436±0.0091 | bc | 0.7830±0.0021 | a | 0.7674±0.0045 | ab |
| **TCARI** | 0.4407±0.0090 | bc | 0.5071±0.0192 | a | 0.4906±0.0128 | ab | 0.4107±0.0125 | c | 0.4145±0.0105 | c |
| **TCARI / OSAVI** | 0.5560±0.0115 | a | 0.7419±0.0406 | b | 0.6616±0.0240 | b | 0.5195±0.0155 | a | 0.5337±0.0138 | a |
| **TVI** | 59.06±0.6821 | a | 57.23±1.6620 | a | 58.46±1.0837 | a | 55.88±1.1119 | a | 55.95±0.8576 | a |
| **G** | 0.2036±0.0026 | c | 0.2953±0.0247 | a | 0.2615±0.0108 | ab | 0.2010±0.0028 | c | 0.2186±0.0048 | bc |
| **ZTM** | 1.8971±0.0138 | a | 1.6570±0.0420 | b | 1.7085±0.0298 | b | 1.8579±0.0129 | a | 1.8842±0.0159 | a |
| **VOG1** | 1.5268±0.0078 | a | 1.4171±0.0208 | b | 1.4403±0.0163 | b | 1.5036±0.0081 | a | 1.5308±0.0083 | a |
| **CAR** | 0.7070±0.0053 | b | 0.7076±0.0049 | b | 0.7284±0.0080 | ab | 0.7344±0.0114 | a | 0.7314±0.0065 | a |
| **LIC3** | 0.1557±0.0024 | c | 0.1761±0.0064 | a | 0.1690±0.0031 | ab | 0.1534±0.0017 | c | 0.1627±0.0025 | bc |
| **RARS** | 5.1587±0. 0614 | ab | 4.2801±0.1786 | c | 4.4389±0.1203 | c | 5.4139±0.0822 | a | 4.9607±0.0926 | b |
| **PRI515** | -0.2176±0.0019 | b | -0.2052±0.0035 | ab | -0.1894±0.0054 | a | -0.1960±0.0068 | a | -0.1926±0.0034 | a |
| **PRIn** | -0.0233±0.0013 | b | -0.0082±0.0067 | a | -0.0160±0.0021 | ab | -0.0210±0.0011 | b | -0.0205±0.0011 | b |
| **ARI** | -0.6001±0.0244 | c | -0.2711±0.0403 | bc | -0.0603±0.1173 | ab | 0.1819±0.1833 | a | -0.2907±0.0760 | bc |
| **RVSI** | 0.0028±0.0007 | b | 0.0060±0.0008 | a | 0.0047±0.0007 | ab | 0.0028±0.0007 | b | 0.0040±0.0005 | ab |
| **HVI** | 5.4345±0.0693 | a | 4.2532±0.2241 | b | 4.3043±0.1603 | b | 5.3155±0.0615 | a | 5.0158±0.0938 | a |
| **GI** | 2.8306±0.0354 | a | 2.6309±0.0889 | ab | 2.5209±0.0729 | b | 2.5583±0.0785 | b | 2.5206±0.0389 | b |
| **DVI** | 0.6663±0.0059 | a | 0.6080±0.0159 | b | 0.6494±0.0082 | a | 0.6673±0.0062 | a | 0.6490±0.0068 | a |
| **RVI** | 0.1070±0.0013 | b | 0.1674±0.0203 | a | 0.1371±0.0066 | ab | 0.1088±0.0013 | b | 0.1166±0.0022 | b |
| **SAVI** | 0.7781±0.0021 | a | 0.7056±0.0220 | c | 0.7389±0.0084 | bc | 0.7756±0.0019 | a | 0.7616±0.0037 | ab |
| **TSAVI** | 0.8085±0.0020 | a | 0.7387±0.0210 | c | 0.7665±0.0087 | bc | 0.8059±0.0019 | a | 0.7919±0.0036 | ab |
| **PVI** | 0.1540±0.0064 | a | 0.1339±0.0059 | a | 0.1561±0.0078 | a | 0.1554±0.0070 | a | 0.1423±0.0071 | a |
| **NDVI 705** | 0.4274±0.0041 | a | 0.3349±0.0177 | b | 0.3586±0.0113 | b | 0.4169±0.0038 | a | 0.4191±0.0050 | a |
| **mNDVI 705** | 0.5504±0.0144 | a | 0.4239±0.0216 | b | 0.4535±0.0142 | b | 0.5229±0.0049 | a | 0.5364±0.0067 | a |
| **mSR705** | 0.7475±0.0029 | a | 0.7171±0.0087 | c | 0.7259±0.0046 | bc | 0.7498±0.0024 | a | 0.7365±0.0034 | ab |
| **Green NDVI** | 0.4847±0.0044 | ab | 0.4049±0.0177 | c | 0.4506±0.0111 | b | 0.5258±0.0109 | a | 0.4936±0.0073 | ab |
| **VOG2** | -0.0582±0.0011 | b | -0.0452±0.0026 | a | -0.0482±0.0020 | a | -0.0558±0.0012 | b | -0.0592±0.0012 | b |
| **VOG 3** | -0.0629±0.0012 | b | -0.0482±0.0029 | a | -0.0516±0.0023 | a | -0.0600±0.0013 | b | -0.0639±0.0013 | b |
| **HNDVI** | 0.8164±0.0021 | a | 0.7457±0.0227 | c | 0.7714±0.0096 | bc | 0.8134±0.0021 | a | 0.8020±0.0035 | ab |
| **MCARI** | 1.2835±0.0244 | a | 1.2125±0.0592 | ab | 1.2311±0.0334 | ab | 1.3059±0.0229 | a | 1.1391±0.0327 | b |
| **WBI** | 3.3821±0.0166 | a | 3.2325±0.0590 | b | 3.2167±0.0420 | b | 3.2872±0.0351 | ab | 3.2611±0.0177 | ab |
| **FWBI1** | 1.0254±0.0018 | ab | 1.0353±0.0026 | a | 1.0211±0.0040 | b | 1.0335±0.0030 | a | 1.0279±0.0024 | ab |
| **FWBI2** | 1.0273±0.0022 | ab | 1.0383±0.0027 | a | 1.0221±0.0045 | b | 1.0375±0.0031 | a | 1.0302±0.0026 | ab |
| **VARI Green** | 0.0810±0.0034 | a | 0.0328±0.0072 | abc | 0.0043±0.0193 | bc | -0.0175±0.0219 | c | 0.0390±0.0088 | ab |
| **SRPI** | 1.3251±0.0118 | a | 1.1573±0.0419 | b | 1.1707±0.0269 | b | 1.2970±0.0089 | a | 1.2675±0.0101 | a |
| **Lomin** | 0.0790±0.0013 | b | 0.1208±0.0146 | a | 0.1021±0.0051 | ab | 0.0807±0.0015 | b | 0.0843±0.0017 | b |
| **RGRcn** | 0.6305±0.0035 | b | 0.6974±0.0251 | a | 0.7190±0.0202 | a | 0.6899±0.0131 | ab | 0.6736±0.0073 | ab |
| **AI** | 0.9305±0.0243 | b | 0.9435±0.0534 | b | 1.0283±0.1035 | b | 1.6722±0.2989 | a | 1.0525±0.0713 | b |
| **R705/(R717+R491)** | 0.5117±0.0029 | b | 0.5665±0.0103 | a | 0.5521±0.0069 | a | 0.5193±0.0024 | b | 0.5151±0.0032 | b |
| **REP** | 36.71±0.2132 | b | 59.24±0.7919 | a | 43.09±1.3703 | b | 37.03±0.1868 | b | 37.98±0.3716 | b |
